# Maternal Diet Quality and Multivitamin Intake During Pregnancy Interact in the Association with Offspring Neurodevelopment at 2 Years of Age

**DOI:** 10.3390/nu17122020

**Published:** 2025-06-17

**Authors:** Yamei Yu, Han Liu, Cindy Feng, Jean R. Seguin, Isabelle S. Hardy, Wenguang Sun, Tim Ramsay, Julian Little, Beth Potter, Marie-Noëlle Simard, Gina Muckle, Andrea MacLeod, William D. Fraser, Lise Dubois

**Affiliations:** 1 School of Epidemiology and Public Health, Faculty of Medicine, University of Ottawa, Ottawa, ON K1N 6N5, Canada; tramsay@ohri.ca (T.R.); jlittle@uottawa.ca (J.L.); beth.potter@uottawa.ca (B.P.); ldubois@uottawa.ca (L.D.); 2 Obstetrics and Gynecology Hospital of Fudan University, Shanghai 200011, China; liuhan1106@126.com; 3 Department of Community Health and Epidemiology, Faculty of Medicine, Dalhousie University, Halifax, NS B3H 4R2, Canada; cindy.feng@uottawa.ca; 4 Department of Psychiatry and Addictology, University of Montreal, Montreal, QC H3T 1J4, Canada; jean.seguin@umontreal.ca; 5 Department of Obstetrics and Gynecology, Faculty of Medicine and Health Sciences, Université de Sherbrooke, Sherbrooke, QC J1H 5N4, Canada; 6 The International Peace Maternity and Child Health Hospital, School of Medicine, Shanghai Jiao Tong University, Shanghai 200030, China; sunwenguang68@126.com; 7 School of Rehabilitation, University of Montreal, Montreal, QC H3N 1X7, Canada; marie-noelle.simard@umontreal.ca; 8 School of Psychology, Laval University, Quebec City, QC G1V 0A6, Canada; gina.muckle@psy.ulaval.ca; 9 Department of Communication Sciences & Disorders, University of Alberta, Edmonton, AB T6G 2G4, Canada; andrea.a.n.macleod@ualberta.ca

**Keywords:** prenatal nutrition, Healthy Eating Index, multivitamins, child neurodevelopment

## Abstract

**Objective:** To comprehensively evaluate the interaction between diet quality and multivitamin intake during pregnancy on offspring neurodevelopment. **Methods:** This analysis was grounded in mother-child dyads from the 3D Cohort Study in Quebec, Canada. Among the 2366 participants initially enrolled in the 3D study, 1535 women successfully completed the 3-day food record during 20–24 weeks of gestation. A Canadian adaptation of the Healthy Eating Index (HEI-C) 2010 was used to quantify diet quality. The total HEI-C score was dichotomized into low and high diet quality by median split. Cognitive and motor development in childhood were assessed using the Bayley Scales of Infant and Toddler Development, Third Edition (Bayley-III). Language abilities were measured using the toddler short-form version of the MacArthur–Bates Communicative Development Inventories (MCDI) questionnaire, administered in either English or French. After excluding participants with missing covariate data, cognitive, motor, and language development scores at 2 years of age were available for 1066, 1040, and 981 children, respectively. Multiple linear regression models were employed to calculate adjusted effect estimates. The interaction on an additive scale was assessed by incorporating a product term into the linear regression model. **Results:** Statistically significant interactions were detected between diet quality and multivitamin intake in relation to the cognitive and language development outcomes of the offspring (interaction *p*-values were 0.018 and 0.023, respectively). The lowest cognitive and language scores were observed in the group of women who neither took multivitamins nor maintained a high-quality diet. Among women not taking multivitamins, a high-quality diet was associated with improved offspring cognitive and language scores (mean difference [95% CI] = 4.2 [0.1, 8.2], *p* = 0.04; and 11.3 [3.1, 19.5], *p* = 0.01, respectively). However, among women taking multivitamins, no such associations were identified. Conversely, in participants with a low-quality diet, multivitamin intake was associated with a 3.0-point increase in cognitive composite scores (95% CI: 0.3, 5.8, *p* = 0.03), but this was not the case for those with a high-quality diet. No statistically significant interactions were observed between maternal diet quality and multivitamin intake for motor development outcomes. **Conclusions:** Adequate nutritional supply during pregnancy, achieved either through a high-quality diet or multivitamin supplementation, is fundamental for the neurodevelopment of children.

## 1. Introduction

The first 1000 days of life, which include pregnancy and two years postpartum, is a time when the brain forms and develops rapidly and lays the foundation of neurodevelopment across the lifespan. During this crucial early stage, proliferation, migration, aggregation, and myelination of the neurons form the architecture of the brain and provide the foundation for cognitive, motor, and language functions later in life [1]. Particularly, throughout the third trimester, the structure of the brain changes from a smooth bilobed shape to a more complex one with gyrations and sulcations that resembles the adult brain [2,3]. The early period of neurodevelopment represents a time of great opportunity and vulnerability for future educational attainment and behaviors at the individual level, as well as overall development and economic growth at the population level [4].

Proper nutrition during pregnancy provides fundamental building blocks for the developing brain. The brain is vulnerable to damage if the nutrients required to support its growth are insufficient [5]. Research on the Great Dutch Famine revealed that individuals who were exposed to the famine in utero exhibited smaller brain volumes and reduced perfusion in later life [6,7]. In addition to energy and macronutrient deficiencies characterized in the famine, micronutrients, including zinc, iron, choline, B-vitamins, and iodine, were also found to be essential for brain growth [8].

Pregnant women generally are highly recommended to get essential nutrients through a good-quality diet. When getting the nutrition supply primarily from the diet is pragmatically unattainable, multivitamin supplements may also be considered as an alternative [9]. Diet quality can be assessed by measuring compliance with dietary guidelines [10]. Despite increasing interest in the associations between maternal diet quality during pregnancy and childhood neurodevelopment, to date, there were few studies quantifying this association directly [11,12]. Most previous studies used components of a good-quality diet, such as fish, seafood, fruit intake, certain nutrients, or data-driven dietary pattern, as an approximation of diet quality [11]. A U.S. cohort study demonstrated that high maternal Mediterranean Diet Scores (6–9) during pregnancy, compared with low scores (0–3), were associated with improved intelligence and fewer metacognitive problems in mid-childhood [13]. Additionally, maternal fourth quartile (Q4) vs. first quartile (Q1) Alternate Healthy Eating Index scores were linked to better visual–spatial skills, verbal intelligence, and executive function in offspring. A longitudinal French cohort study reported that higher nutrient intake scores calculated using the Probability of Adequate Nutrient Intake Based Diet Quality Index were correlated with enhanced overall development in children aged 1 to 3.5 years [14]. Furthermore, a Norwegian study observed an inverse association between maternal pregnancy diet quality (measured by the Prenatal Diet Quality Index) and the diagnosis of Attention Deficit Hyperactivity Disorder in children at 8 years of age [15]. However, interventional studies using multivitamin supplementation did not demonstrate positive effects in improving offspring cognitive outcomes [12]. The discrepancy between these results and the findings of observational and intervention studies could possibly be explained by a modifying effect of diet quality (and/or underlying nutritional status) on the association between multivitamin intake and neurodevelopment as both diet and multivitamins provide nutrients during pregnancy. However, no studies have measured the potential interaction between the two exposures. Our hypothesis is that the association between maternal diet quality and child neurodevelopmental outcomes is modified by multivitamin intake during pregnancy. Specifically, among women with poor diet quality, multivitamin supplementation may exert a more pronounced beneficial effect on neurodevelopment. In contrast, in women with high diet quality, the incremental benefit of multivitamins may be blunted or neutral as their diet already provides adequate nutrients.

Therefore, the objective of this paper is to evaluate diet quality and multivitamin intake during pregnancy, and their interactions, in relation to neurodevelopmental outcomes in the offspring.

## 2. Materials and Methods

### 2.1. Design, Setting, and Participants

**Figure 1:**
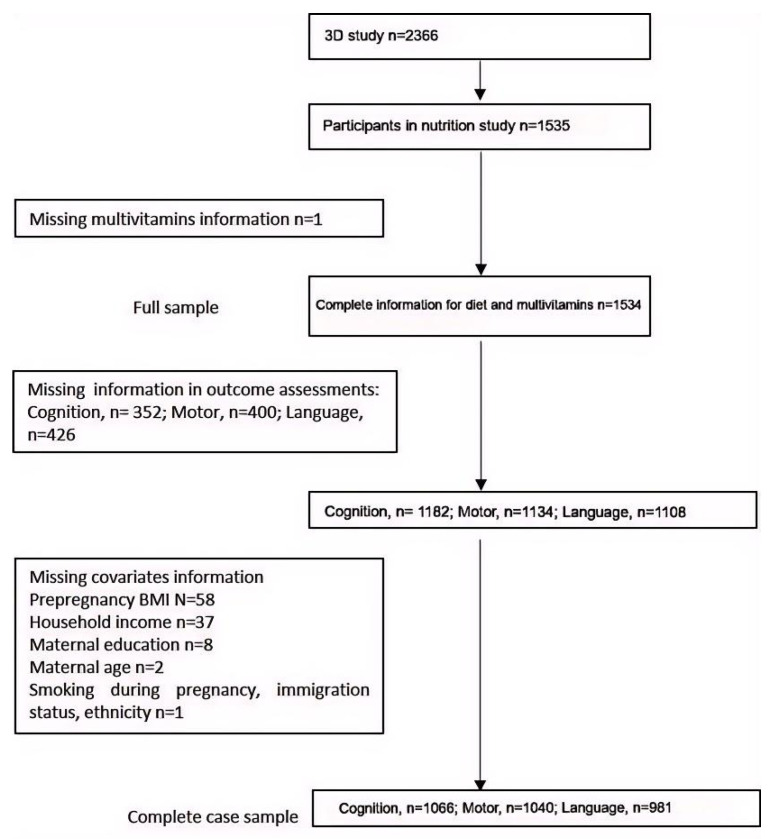
Flow chart for study participants.

This study was based on the 3D Prospective Cohort Study in Quebec, Canada. The protocol of this study and the methods used for dietary data collection and processing have been described in detail elsewhere [9,16,17]. Between 2010 and 2012, 2366 pregnant women from the province of Quebec were recruited in the first trimester of pregnancy. Extensive interviews were conducted in the first, second, and third trimester of pregnancy and postpartum at 3 months, 1 year, and 2 years (see Figure 1). All participants signed written informed consents.

### 2.2. Exposures and Measurements

Women were asked to complete a 3-day food record between 20 and 24 weeks of gestation. Information on dietary intake and use of multivitamins in pregnancy was available for 1534 women. Using the dietary information obtained from the 3-day food records, a Canadian adaptation of the Healthy Eating Index (HEI-C) 2010 was used to quantify diet quality. The HEI-C measures compliance with the Canadian Food Guide recommendations [18] by scoring intakes of eight food groups that should be consumed in adequate amounts including total fruits and vegetables, whole fruit, greens and beans, whole grains, dairy, total protein foods, seafood and plant proteins, fatty acids, and three that should be consumed in moderate amounts including refined grains, sodium, empty calories, resulting in a total score of 100, with higher total scores reflecting higher-quality diets [10]. This index has been validated for use in the general adult population [17] and in the pregnant women of this study. Details of the methods used to code data and food groups for the nutritional analysis of the 3D Cohort Study have been described elsewhere [19]. Briefly, the energy, macronutrients, and micronutrients of each food item were compiled by trained nutritionists using the Food Processor software (version 10.13.1, ESHA Research, Inc., Salem, MA, USA), which was linked to the Canadian Nutrient File [20]. Food items were also classified into four food groups and 30 sub-groups using the food Classification system developed by Health Canada [21]. Mixed dishes were decomposed into food group servings according to the Food Patterns Equivalents Database developed by U.S. Department of Agriculture [22]. This information was used to calculate the scores for each component of the HEI-C, which were compiled to obtain the total HEI-C score. The total HEI-C score was further dichotomized into low and high diet quality by median split.

Multivitamin supplement intake was captured using the maternal medication logs (MML) administered by research nurses during study visits in each trimester of pregnancy and at delivery. The nutritional information of each multivitamin was confirmed by trained nutritionists using Health Canada’s Licensed Natural Health Products Database [23] and each company’s product label and website. For this study, use of multivitamins in pregnancy was considered as a dichotomous variable based on the information stated in the MML of either “did not take multivitamins” or “took multivitamins”.

### 2.3. Outcomes and Measurements

Neurodevelopmental assessments were performed on the children around two years of age by trained research personnel who were unaware of the exposures of this study and covariate information. Cognitive and motor (fine and gross) scales of childhood development were assessed using the Bayley Scales of Infant and Toddler Development, 3rd edition (Bayley-III scale). The Bayley-III is a validated and standardized developmental assessment for children aged 1–42 months with established test–retest reliability and internal consistency as well as convergent and divergent validity [24]. Each scale consists of a series of developmental play tasks. The cognitive scale assesses cognitive processes like memory, exploration, manipulation, and sensorimotor development. The motor scale evaluates the quality of movement, sensory integration, perceptual–motor integration, prehension, and other milestones. Scale-specific raw scores were converted to scaled scores and to composite scores according to the score distribution in normally developed children of the same age. For the fine and gross motor subtests, only scaled scores were available. Means were set at 10 and 100 with a standard deviation of 3 and 15 for the scaled scores (fine and gross motor) and the composite scores (cognitive and motor), respectively. In this study, the intra-class correlation coefficient among trainees for the Bayley-III scale was above 0.90.

To measure language abilities, this study focused on early word production via a standard parent-report task. Specifically, the toddler short-form versions of MacArthur–Bates Communicative Development Inventories (MCDI) questionnaire administered either in English or French were used. This questionnaire is a 100-word checklist that focuses on words commonly used in the lives of young children. Parents were asked to identify the words on the list that their child says, even if the pronunciation does not match the adult target. The English MacArthur–Bates toddler short form has established reliability as well as content and concurrent validity. A French version of the short form has been adapted for French-speaking children in Québec using the approach described by Fenson et al. [25] and in our previous study [26].

### 2.4. Statistical Analyses

Means and standard deviations (SDs) of the neurodevelopment scores were calculated. Univariate and multivariable linear regression models were used to calculate crude and adjusted effect estimates (mean differences, MD) and 95% confidence intervals (CIs). Interaction on an additive scale was assessed by adding a product term to the linear regression model [27]. Variance inflation factors (VIFs) were used to test for multicollinearity in multiple linear regression models. No multicollinearity was observed in the models using a cut-off VIF value of 10. A directed acyclic graph (DAG, Supplementary Figure S1) was used to select potential confounding variables in the adjusted model to estimate the association between maternal nutrition during pregnancy and neurodevelopment outcomes of the children [28]. Maternal characteristics including age, energy intake, pre-pregnancy body mass index (BMI), education, smoking, marital status, immigration status, ethnicity, parity, family income, and children’s characteristics including biological sex and age at neurodevelopment measurement were considered as confounders. As a sensitivity analysis, multiple imputation according to the Markov chain Monte Carlo method was used to input missing information in covariates by creating and pooling 20 imputed data sets for analyses based on “missing at random” assumption [29]. As omega-3 fatty acid supplementation might have a favorable effect on cognitive outcomes in pregnancy, to differentiate the effects of micronutrients versus omega-3 fatty acids on offspring neurodevelopment in our study, we further tested whether omega-3 supplementation was a potential confounder for the observed association between multivitamin supplementation and neurodevelopment by examining (1) the association between omega-3 supplementation and multivitamins supplementation; (2) the association between omega-3 supplementation and neurodevelopment in the whole-study samples; (3) the association between omega-3 supplementation and neurodevelopment in the group with lower diet quality.

All statistical analyses were performed using Statistical Analysis System software version 9.4 (SAS v9.4; SAS Institute Inc., Cary, NC, USA). A two-sided *p* < 0.05 was set as the level of statistical significance. For interaction terms statistically significance, an alpha level of 0.10 was considered.

## 3. Results

### 3.1. Study Characteristics According to Diet Quality and Multivitamin Intake

Out of 2366 participants enrolled in the 3D study, 1535 women completed the 3-day food record and were eligible for study inclusion. One additional participant was excluded due to missing information on multivitamin consumption in pregnancy, leaving 1534 women for inclusion in analysis (full sample). The HEI-C score showed a median of 62.7 (interquartile range [IQR], 55.2–70.6), with a minimum of 28.8 and maximum of 95.6. Diet quality was categorized into low and high groups by dichotomizing the total HEI-C score at the median. As shown in Table 1, compared with participants with low diet quality, participants with high diet quality were older, more likely to have completed university studies, and had higher household income. They were also less likely to be overweight or obese and to smoke. Participants who took multivitamins and those who did not take multivitamins had comparable characteristics, except that those who did not take multivitamins had a higher rate of smoking during pregnancy. The proportion of participants who reported smoking in pregnancy was higher in participants not taking multivitamins (20.5% vs. 12.5%). Children’s MCDI scores at 2 years of age were significantly higher among those whose mothers had a high-quality diet during pregnancy compared to those with a low-quality diet. The multivitamins consumed were predominantly prenatal supplements. Notably, 59% of non-multivitamin users exhibited low diet quality, compared with 49% of multivitamin users. The use of multivitamins in each trimester has been provided in Supplementary Table S1. Overall, 89.5% of participants took multivitamins during one to three trimesters of pregnancy, while 10.5% did not use them in any trimester. Among those who used multivitamins, 96.3% took them daily, 1.9% took them 4–6 times per week, and 1.8% took them 1–3 times per week.

### 3.2. Interactions Between Diet Quality and Multivitamin Intake During Pregnancy on Child Neurodevelopment

Cognitive, motor, and language development scores at 2 years of age were available for 1182, 1134, and 1108 children, respectively. After excluding participants with missing data on covariates, 1066, 1040, and 981 remained for complete case analysis (Figure 1). The characteristics of participants for complete case analysis were very similar to those in the full sample (Supplementary Table S2).

As shown in Table 2, there were statistically significant interactions between diet quality and multivitamin intake during pregnancy for the outcome of cognitive development of children at 2 years of age measured by Bayley-III (*p* for interaction = 0.018 in the adjusted model). The lowest cognitive composite score (mean [SD] = 96.9 [11.4]) was found in the group with low diet quality and no multivitamin use. After adjusting for multiple potential confounding variables, having a high diet quality during pregnancy was significantly associated with higher cognitive composite scores in the offspring among women who did not take multivitamins during pregnancy (MD [95% CI] = 4.2 [0.1, 8.2], *p* = 0.04) but not among women who took multivitamins (MD [95% CI] = −1 [−2.4, 0.4], *p* = 0.16). Similarly, taking multivitamins during pregnancy was associated with higher cognitive composite scores in the offspring among women who had a low diet quality during pregnancy (MD [95% CI] = 3 [0.3, 5.8], *p* = 0.03) but not in those who had a high diet quality (MD [95% CI] = −2.1 [−5.4, 1.1], *p* = 0.19).

Similarly, there were statistically significant interactions between diet quality and multivitamin intake during pregnancy for the outcome of language development of children at 2 years of age (*p* for interaction = 0.008 and 0.023, respectively, in unadjusted and adjusted models). In adjusted models, among women who did not take multivitamins during pregnancy, the MacArthur–Bates language score was 11.3 points (95% CI, 3.1, 19.5; *p* = 0.01) higher in children of women with a high pregnancy diet quality, compared with those of women with a low diet quality, while no such association was observed among women who took multivitamins (MD [95% CI] = 1.2 [−1.6, 4.1], *p* = 0.39). After adjustment, no statistically significant differences in offspring MacArthur–Bates language scores were found between multivitamin users and non-users in either the low (MD [95% CI] = 4.9 [−0.7, 10.4], *p* = 0.09) or high (MD [95% CI] = −5.2 [−11.9, 1.5], *p* = 0.13) diet quality groups.

As shown in Table 3, after adjustment, there were no statistically significant interactions between diet quality and multivitamin intake during pregnancy for motor development outcomes of children at 2 years of age. The regression coefficients for the models have been shown in Supplementary Tables S3-1 and S3-2.

In the sensitivity analyses with imputation of missing values in covariates, similar results were found (see Supplementary Table S4, *p* for interaction = 0.018 and 0.005, respectively, in adjusted models for cognitive and language development, respectively).

We found no statistically significant associations in these hypotheses: no association between omega-3 supplementation and multivitamin supplementation; no association between omega-3 supplementation and neurodevelopment. These results indicated that the effect of multivitamins that we observed in this study is not due to omega-3 supplementation (see Supplementary Tables S5 and S6).

## 4. Discussion

The results of this study demonstrate that there is a significant interaction between diet quality and multivitamin intake in pregnancy in the association with the cognitive and language development of children at 2 years of age. High diet quality during pregnancy was associated with better cognitive and language development in the offspring among women who did not take multivitamins. No such associations were found in those who took multivitamins. Similarly, taking multivitamins during pregnancy was associated with better cognitive development in the offspring among women with low diet quality. No such associations were found among those with a higher diet quality. The lowest cognitive and language development results were observed in the group of children whose mothers ate a lower-quality diet and did not take multivitamins during pregnancy. This group of women were lacking in the quality of nutrition supply from food and supplement sources compared to those in the other three groups (Supplementary Table S7). To our knowledge, no previous studies have reported this interaction.

Women with poor dietary patterns may exhibit greater baseline nutritional deficiencies, rendering multivitamins more impactful in improving cognitive outcomes. In nutritionally well-supported women, multivitamins likely serve as “nutritional insurance” rather than direct drivers of additional cognitive enhancements. Additionally, high diet quality is frequently associated with other protective lifestyle behaviors, which may attenuate the observed effects of multivitamins within this subgroup. These findings highlight that the interaction between diet quality and multivitamin use is context-dependent, shaped by baseline nutritional status, nutrient-specific requirements across developmental domains, and potential ceiling effects of supplementation in well-nourished populations. Further investigation into nutrient-specific biomarkers and longitudinal developmental trajectories would help disentangle these complex mechanisms.

Assuming a causal relationship, several mechanisms could explain the association between early nutrition and children’s neurodevelopment (“total effect”). First, high-quality nutrition directly provides nutrients that work as fundamental building blocks for brain growth and development (“direct effect”) [30,31]. Animal studies have shown that maternal diets deficient in micronutrients could lead to hippocampal learning deficits [32], which can adversely impact several abilities (which would include cognition and language in humans) [33]. Second, the relationship between the quality of nutrition during pregnancy and offspring neurodevelopment could be mediated by birth weight, preterm birth, or by children’s diet (“indirect effect”, see Supplementary Figure S1). Indeed, micronutrient supplementation in pregnancy has been associated with a small reduction in the rates of preterm birth, small for gestational age, and low birth weight [34]. Maternal diet quality is correlated with the diet quality of their children. A combination of improved perinatal outcomes and better diet quality during childhood could also partly explain the association between adequate nutrition during pregnancy and improved offspring neurodevelopment [35]. A mediation analysis could help to determine the contribution of each pathway in a potential causal relationship [36]. However, insufficient power precluded us from performing mediation analysis in the study sample due to the small portion of pregnant women who did not take multivitamins. Therefore, this study only provides estimates of the global association between prenatal nutrition and offspring neurodevelopment (“total effect”). Future studies should formally decompose these effects via mediation analysis to quantify the proportion mediated by gestational age, birth weight, and children’s diet as compared to direct biological pathways.

Our study suggests that prenatal multivitamin supplementation or maintaining a high-quality diet during pregnancy may promote offspring neurodevelopmental outcomes. This concurs with the results from a multivitamin supplementation study in Indonesia [37]. Although there were no effects in the overall study population, among undernourished women, children in the multivitamin group showed better cognitive abilities than in the control group. In a double-blind, randomized, controlled trial in rural China, multivitamin supplementation led to 1.2 points increase in the mental development scales of the BSID (second version) of children reported at 1 year of age [38]. Another study in rural China reported that antenatal multiple micronutrient supplementation was associated with increased adolescent intellectual development at 12 years [39]. To date, a very limited number of studies have evaluated the effect of multivitamin supplementation in pregnancy on offspring neurodevelopment thus far, and there are even less studies for diet quality interventions [12]. More studies on this question are needed and should include an evaluation of the baseline nutrition status of the population.

The results of this study demonstrate a significant interaction between diet quality and multivitamin intake during pregnancy in relation to the cognitive and language development of children at 2 years of age but not with motor development. Several factors may contribute to these findings. The fetal stage witnesses a remarkable surge in brain development, with neuronal proliferation and synapse formation peaking during the second and third trimesters of pregnancy, thereby establishing the foundation for cognitive functions. In contrast, the myelination of motor coordination centers, particularly the cerebellum, predominantly occurs within 6 to 12 months after birth [40]. This distinct developmental timeline suggests that prenatal nutrition exerts a “programming effect” on cognitive development as the fetal brain is highly sensitive to nutrient availability during its rapid growth phase. Conversely, motor development appears to be more influenced by postnatal factors, such as nutritional intake and physical activity levels, which play crucial roles in refining motor skills after birth. This disparity in developmental regulation likely accounts, at least in part, for the lack of association observed between maternal nutrition during pregnancy and children’s motor skill development. Future research should focus on improving study design, employing more precise measurement tools, controlling for additional confounding factors, and exploring in greater depth the relationship between prenatal diet quality and various aspects of children’s development.

The World Health Organization (WHO) currently recommends iron and folic acid supplementation for women during pregnancy as part of routine antenatal care. However, multivitamin supplementation has not been recommended. Despite Health Canada’s recommendation of daily multivitamins supplements and the provision of free multivitamins for low-income pregnant women as part of the OLO program in the province of Quebec [41], 10.5% of the women in this cohort did not take any multivitamins during pregnancy. This is particularly concerning as our study showed that taking multivitamins might mitigate the potential harm of nutrient deficiency on offspring neurodevelopment in women with a poor diet. Future studies evaluating the barriers to multivitamin use in pregnancy are needed to inform targeted interventions in women with low diet quality at the start of pregnancy.

Our study has several strengths. Most previous studies evaluated exposure to single nutrients including zinc, iron, choline, B-vitamins, and iodine rather than multivitamin intake [8,42]. It is worth noting that nutrient deficiencies rarely occur individually during pregnancy [43], and most RCTs of single nutrient supplementation did not identify beneficial effects [12]. The use of the Healthy Eating Index as a measurement of overall diet quality in this study is more relevant for public health interventions than single nutrient studies. Our study is the first to investigate the interaction between maternal dietary quality and prenatal multivitamin intake on offspring neurodevelopment. Although the percentage of women not taking multivitamins in this study is low, the high quality and large sample of the cohort provided sufficient power to conduct analyses with a large set of covariates. Furthermore, the 3-day food record dietary assessment method used in this study is recognized as one of the most accurate tools for dietary assessment and is less prone to memory biases than food frequency questionnaires or 24 h recalls [44].

Our study also has several limitations. Although we identified potential confounding variables using DAG, causal relationship cannot be established in an observational study. Further interventional studies such as RCTs are needed to strengthen the argument for causality. Additionally, unmeasured or inadequately measured factors could influence the results and introduce bias. Participants with missing information in the covariates or outcomes were excluded in the main analysis, which could possibly lead to selection bias. However, our sensitivity analysis using multiple imputation generated similar trends, indicating that the risk of selection bias specifically due to missing data is low. Although the exposures and outcomes were measured with validated tools, misclassification remains possible owing to the difficulty of measuring diet and neurodevelopment. Research personnel who conducted the measurements of neurodevelopment were blinded to the exposure or other background information of the children and family. As a result, the misclassification is more likely to be nondifferential across exposure groups and would bias effect estimates towards the null hypothesis [45]. It is important to note that food records may not adequately reflect long-term dietary patterns or seasonal variations in food availability and consumption. Dichotomizing diet quality at the median, while a reasonable choice for simplicity and statistical power, results in a loss of information from the original continuous score. The suboptimal neurodevelopmental follow-up rate is one of the study’s limitations. The distribution of our study population was skewed towards that of an older high socio-economic status group and is not a representative sample of the population of Canadian pregnant women [16,19]. However, a previous study showed that the effect of selection bias due to the higher socioeconomic status on exposure–outcome associations could be limited [46]. Lastly, our study only captures diet in the second/third trimester of the pregnancy. However, other studies have shown that diet quality changes little across trimesters [47].

## 5. Conclusions

There is a statistically significant interaction between diet quality and multivitamin intake during pregnancy in association with cognitive and language development of children at 2 years of age. The efforts to improve offspring neurodevelopment outcomes through nutritional intervention should specifically target women with poor diet quality and not taking multivitamins.

## Figures and Tables

**Table 1:** Participant characteristics according to diet quality and multivitamin intake (n = 1534).

Characteristics	Low Diet Quality ^1^ (HEI-C Score < 62.7)	High Diet Quality ^1^ (HEI-C Score ≥ 62.7)		No Multivitamin Use	Multivitamin Use	
	n (%)/Mean ± SD	n (%)/Mean ± SD	*p*	n (%)/Mean ± SD	n (%)/Mean ± SD	*p*
Total	767	767		161	1373	
Mother’s age (years)			<0.001			0.74
<25	61 (8.0)	24 (3.1)		11 (6.8)	74 (5.4)	
25–<35	563 (73.6)	565 (73.8)		116 (72.1)	1012 (73.9)	
≥35	141 (18.4)	177 (23.1)		34 (21.1)	284 (20.7)	
Maternal education			<0.001			0.78
Secondary school or less	65 (8.5)	29 (3.8)		12 (7.5)	82 (6.0)	
College	231 (30.4)	159 (20.8)		37 (23.0)	353 (25.9)	
Undergraduate university degree	298 (39.2)	330 (43.3)		69 (42.8)	559 (41.0)	
Graduate university studies	167 (21.9)	245 (32.1)		43 (26.7)	369 (27.1)	
Household income (CAD)			<0.001			0.13
<30,000	65 (8.8)	51 (6.9)		14 (9.0)	102 (7.7)	
30,000–59,999	141 (19.0)	113 (15.3)		38 (24.3)	216 (16.3)	
60,000–79,999	150 (20.2)	112 (15.2)		24 (15.4)	238 (18.0)	
80,000–99,999	167 (22.5)	165 (22.4)		31 (19.9)	301 (22.8)	
≥100,000	219 (29.5)	296 (40.2)		49 (31.4)	466 (35.2)	
Marital status			0.12			0.96
Married	306 (40.0)	308 (40.2)		65 (40.4)	549 (40.0)	
Common law/partner	420 (54.8)	435 (56.7)		90 (55.9)	765 (55.8)	
Others	40 (5.2)	24 (3.1)		6 (3.7)	58 (4.2)	
Pre-pregnancy BMI (kg/m^2^)			<0.001			0.79
Underweight (<18.5)	36 (4.9)	54 (7.4)		10 (6.5)	80 (6.1)	
Normal weight (18.5–24.9)	442 (60.5)	511 (70.4)		98 (63.6)	855 (65.6)	
Overweight (25–29.9)	143 (19.6)	102 (14.0)		30 (19.5)	215 (16.5)	
Obese (≥30)	110 (15.0)	59 (8.2)		16 (10.4)	153 (11.8)	
Parity			<0.001			0.46
0	408 (53.2)	482 (62.8)		89 (55.3)	801 (58.3)	
≥1	359 (46.8)	285 (37.2)		72 (44.7)	572 (41.7)	
Mother born in Canada			0.97			0.17
No	217 (28.4)	217 (28.3)		53 (32.9)	381 (27.8)	
Yes	548 (71.6)	550 (71.7)		108 (67.1)	990 (72.2)	
Caucasian			0.96			0.70
No	152 (19.9)	153 (20.0)		30 (18.8)	275 (20.1)	
Yes	613 (80.1)	613 (80.0)		130 (81.2)	1096 (79.9)	
Smoking during pregnancy			0.007			0.005
No	646 (84.3)	681 (89.0)		128 (79.5)	1199 (87.5)	
Yes	120 (15.7)	84 (11.0)		33 (20.5)	171 (12.5)	
Sex of the child			0.18			0.73
Female	391 (51.4)	368 (48.0)		77 (48.4)	682 (49.9)	
Male	369 (48.6)	398 (52.0)		82 (51.6)	685 (50.1)	
Child neurodevelopment assessment age (months)	25.5 ± 2.3	25.4 ± 2.1	0.32	25.5 ± 2.1	25.4 ± 2.2	0.78
Bayley-III cognitive scores	100.2 ± 11.2	100.8 ± 11.6	0.36	99.5 ± 12.0	100.6 ± 11.3	0.32
Bayley-III motor scores	101.7 ± 12.3	101.8 ± 12.1	0.97	103.8 ± 13.0	101.5 ± 12.1	0.07
MCDI language scores	53.6 ± 23.0	56.8 ± 24.1	0.02	52.9 ± 24.7	55.5 ± 23.5	0.26
Maternal calorie intake (kcal per day)	2166.5 ± 471.3	2224.3 ± 451.4	0.01	2178.0 ± 441.5	2197.5 ± 464.8	0.61

HEI-C: Healthy Eating Index—Canada; SD: standard deviation; BMI: body mass index; ^1^ low and high diet quality was defined by dichotomizing the total HEI-C score by median split.

**Table 2:** Interaction between diet quality and multivitamin intake during pregnancy on cognitive and language development of children at 2 years of age.

	No Multivitamin Use		Multivitamin Use		MD (95% CI) for Multivitamins Within Strata of Diet Quality	*p* for Interaction
	Mean (SD), n	MD (95% CI), *p*	Mean (SD), n	MD (95% CI), *p*		
Cognitive, unadjusted						0.007
Low diet quality	96.9 (11.4), 69	Reference	100.9 (11.4), 476	4 (1.2, 6.9), *p* = 0.01 ^1^	4 (1.2, 6.9), *p* = 0.01 ^1^	
High diet quality	102.8 (11.4), 47	5.9 (1.7, 10.1), *p* = 0.01 ^1^	100.6 (11.4), 491	3.7 (0.9, 6.6), *p* = 0.01 ^1^	−2.1 (−5.5, 1.3), *p* = 0.22 ^2^	
MD (95% CI) for diet quality within strata of multivitamins		5.9 (1.7, 10.1), *p* = 0.01 ^1^		0.3 (−1.1, 1.7), *p* = 0.7 ^3^		
Cognitive, adjusted *						0.018
Low diet quality	94.9 (13.3), 69	Reference	97.9 (22.3), 476	3 (0.3, 5.8), *p* = 0.03 ^1^	3 (0.3, 5.8), *p* = 0.03 ^1^	
High diet quality	99 (12.5), 47	4.2 (0.1, 8.2), *p* = 0.04 ^1^	96.9 (23.8), 491	2 (−0.7, 4.8), *p* = 0.15 ^1^	−2.1 (−5.4, 1.1), *p* = 0.19 ^2^	
MD (95% CI) for diet quality within strata of multivitamins		4.2 (0.1, 8.2), *p* = 0.04 ^1^		−1 (−2.4, 0.4), *p* = 0.16 ^3^		
Language, unadjusted						0.008
Low diet quality	47.3 (23.2), 64	Reference	54.6 (23.2), 434	7.3 (1.2, 13.4), *p* = 0.02 ^1^	7.3 (1.2, 13.4), *p* = 0.02 ^1^	
High diet quality	61.9 (23.2), 42	14.6 (5.6, 23.6), *p* < 0.0005 ^1^	56.2 (23.2), 441	8.9 (2.8, 15), *p* < 0.0005 ^1^	−5.7 (−13, 1.6), *p* = 0.02 ^2^	
MD (95% CI) for diet quality within strata of multivitamins		14.6 (5.6, 23.6), *p* < 0.0005 ^1^		1.6 (−1.5, 4.7), *p* = 0.31 ^3^		
Language, adjusted *						0.023
Low diet quality	43.9 (26.1), 64	Reference	48.8 (45), 434	4.9 (−0.7, 10.4), *p* = 0.09 ^1^	4.9 (−0.7, 10.4), *p* = 0.09 ^1^	
High diet quality	55.2 (24.8), 42	11.3 (3.1, 19.5), *p* = 0.01 ^1^	50 (47.8), 441	6.1 (0.5, 11.7), *p* = 0.03 ^1^	−5.2 (−11.9, 1.5), *p* = 0.13 ^2^	
MD (95% CI) for diet quality within strata of multivitamins		11.3 (3.1, 19.5), *p* = 0.01 ^1^		1.2 (−1.6, 4.1), *p* = 0.39 ^3^		

SD: standard deviation; MD: mean difference, denoting for the regression coefficient in the linear regression model. ^1^ Reference: low diet quality and no multivitamin use. ^2^ Reference: high diet quality and no multivitamin use. ^3^ Reference: low diet quality and multivitamin use. * Adjusted for maternal characteristics including age, energy intake, pre-pregnancy body mass index (BMI), education, smoking, marital status, immigration status, ethnicity, parity, family income, and children’s characteristics including biological sex and age at neurodevelopment measurement.

**Table 3:** Interaction between diet quality and multivitamin intake during pregnancy on motor development of children at 2 years of age.

	No Multivitamin Use		Multivitamin Use		MD (95% CI) for Multivitamins Within Strata of Diet Quality	*p* for Interaction
	Mean (SD), n	MD (95% CI), *p*	Mean (SD), n	MD (95% CI), *p*		
Fine motor, unadjusted						0.04
Low diet quality	11.3 (2.6), 65	Reference	11.6 (2.6), 464	0.3 (−0.4, 1), *p* = 0.34 ^1^	0.3 (−0.4, 1), *p* = 0.34 ^1^	
High diet quality	12.3 (2.6), 44	1 (−0.1, 2), *p* = 0.06 ^1^	11.5 (2.6), 467	0.2 (−0.5, 0.9), *p* = 0.63 ^1^	−0.8 (−1.6, 0), *p* = 0.06 ^2^	
MD (95% CI) for diet quality within strata of multivitamins		1 (−0.1, 2), *p* = 0.06 ^1^		−0.2 (−0.5, 0.2), *p* = 0.34 ^3^		
Fine motor, adjusted *						0.17
Low diet quality	11.2 (3.2), 65	Reference	11.3 (5.4), 464	0.2 (−0.5, 0.8), *p* = 0.62 ^1^	0.2 (−0.5, 0.8), *p* = 0.62 ^1^	
High diet quality	11.7 (3), 44	0.5 (−0.5, 1.5), *p* = 0.31 ^1^	11.1 (5.7), 467	−0.1 (−0.7, 0.6), *p* = 0.88 ^1^	−0.6 (−1.4, 0.2), *p* = 0.17 ^2^	
MD (95% CI) for diet quality within strata of multivitamins		0.5 (−0.5, 1.5), *p* = 0.31 ^1^		−0.2 (−0.6, 0.1), *p* = 0.2 ^3^		
Gross motor, unadjusted						0.112
Low diet quality	9 (2.3), 65	Reference	8.9 (2.3), 464	−0.2 (−0.7, 0.4), *p* = 0.6 ^1^	−0.2 (−0.7, 0.4), *p* = 0.60 ^1^	
High diet quality	9.8 (2.3), 44	0.7 (−0.1, 1.6), *p* = 0.09 ^1^	8.9 (2.3), 467	0.7 (−0.2, 1.7), *p* = 0.11 ^1^	−0.9 (−1.6, −0.2), *p* = 0.01 ^2^	
MD (95% CI) for diet quality within strata of multivitamins		0.7 (−0.1, 1.6), *p* = 0.09 ^1^		0 (−0.3, 0.3), *p* = 0.98 ^3^		
Gross motor, adjusted *						0.163
Low diet quality	9 (2.8), 65	Reference	8.8 (4.7), 464	−0.2 (−0.8, 0.4), *p* = 0.43 ^1^	−0.2 (−0.8, 0.4), *p* = 0.43 ^1^	
High diet quality	9.7 (2.6), 44	0.7 (−0.2, 1.5), *p* = 0.13 ^1^	8.8 (4.9), 467	−0.2 (−0.8, 0.4), *p* = 0.49 ^1^	−0.9 (−1.6, −0.2), *p* = 0.01 ^2^	
MD (95% CI) for diet quality within strata of multivitamins		0.7 (−0.2, 1.5), *p* = 0.13 ^1^		0 (−0.3, 0.3), *p* = 0.87 ^3^		

SD: standard deviation; MD: mean difference, denoting for the regression coefficient in the linear regression model. ^1^ Reference: low diet quality and no multivitamin use. ^2^ Reference: high diet quality and no multivitamin use. ^3^ Reference: low diet quality and multivitamin use. * Adjusted for maternal characteristics including age, energy intake, pre-pregnancy body mass index (BMI), education, smoking, marital status, immigration status, ethnicity, parity, family income, and children’s characteristics including biological sex and age at neurodevelopment measurement.

## Data Availability

Data is available from the 3D study committee upon request and approvement. The data are not publicly available due to privacy reason.

## Supplementary Materials

The following supporting information can be downloaded at: https://www.mdpi.com/article/10.3390/nu17122020/s1, Figure S1: Directed acyclic graph (DAG) used to determine minimal sufficient adjustment sets to estimate the association between maternal nutrition and offspring neurodevelopment (‘total effect’). Table S1: Multivitamin intake in each trimester (N = 1534). Table S2: Participant characteristics in the full sample, the complete case sample for the outcomes of child neurodevelopment at 2 years of age in the 3D Cohort Study. Table S3-1: Regression coefficients of the adjusted models for child cognitive and language development at 2 years of age. Table S3-2: Regression coefficients of the adjusted models for fine and gross motor development. Table S4: Sensitivity analyses with imputation of missing values in covariates. Table S5: Association between multivitamin use and omega-3 supplement use. Table S6: Association between taking omega-3 and neurodevelopment outcomes. Table S7: Mean (SD) intake of selected nutrients in the study population.

## Abbreviations

The following abbreviations are used in this manuscript:

HEI-C	Canadian adaptation of the Healthy Eating Index
MML	Maternal medication log
MCDI	MacArthur–Bates Communicative Development Inventories
SD	Standard deviation
MD	Mean difference
CI	Confidence interval
DAG	Directed acyclic graph
BMI	Body mass index
