# Supplementary material for: Maternal Diet Quality and Multivitamin Intake During Pregnancy Interact in the Association with Offspring Neurodevelopment at 2 Years of Age
Source: Nutrients. 2025 Jun 17;17(12):2020. doi: 10.3390/nu17122020 (PMC12196225; doi:10.3390/nu17122020)
Supplement: Supplementary file 1 [file nutrients-17-02020-s001.zip › nutrients-3613381-supplementary.pdf]

Interaction between maternal diet quality and multivitamin intake during pregnancy on offspring neurodevelopment at 2 years of age

Yamei Yu et al.

Supplementary Figure S1 Directed acyclic graph (DAG) used to determine minimal sufficient adjustment sets to estimate the association between maternal nutrition and offspring neurodevelopment ('total effect').

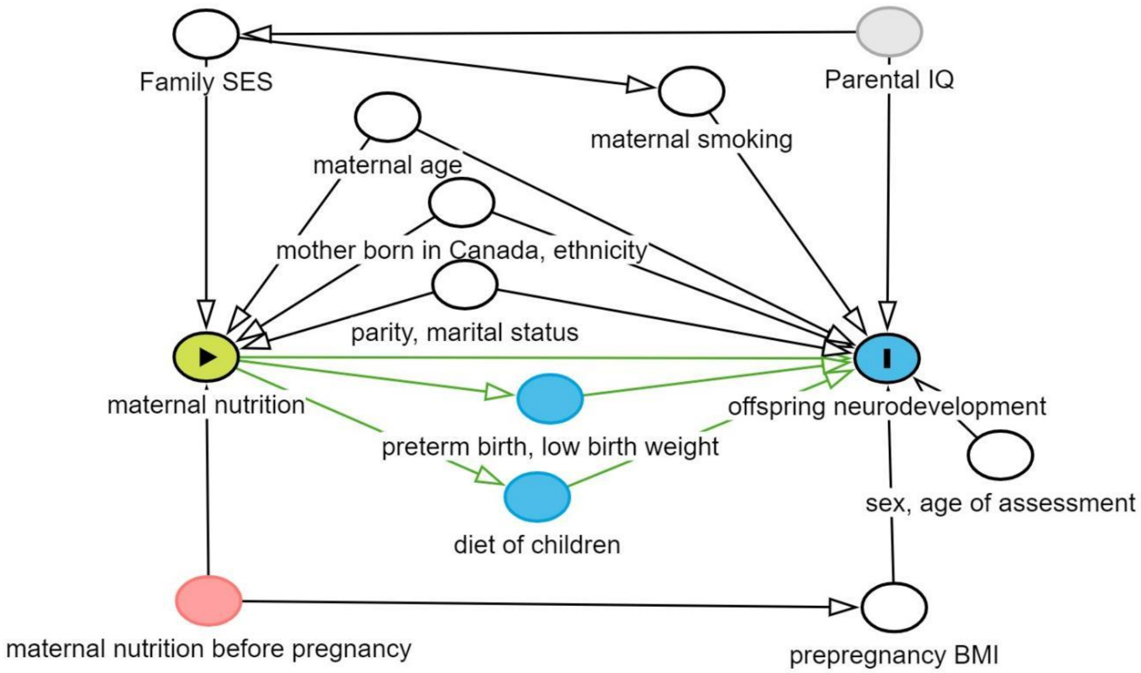

Legend: = exposure; = outcome; = ancestor of outcome; = ancestor of exposure and outcome; = adjusted variables; = unobserved variables; = causal path.

Minimal sufficient adjustment sets used to estimate the total effect of maternal nutrition on offspring neurodevelopment include biological sex, family income, maternal age, maternal education, maternal smoking, mother born in Canada, ethnicity, parity, marital status, and prepregnancy BMI. The relationship between proper nutrition during pregnancy and offspring neurodevelopment could be mediated by birth weight, preterm birth or by children's diet ('indirect effect').

Supplementary Table S1. Multivitamin intake in each trimester (N=1534). [1]

First	Second	Third	Proportion, %
N	N	N	10.5%
N	N	Y	2.2%
N	Y	N	1.0%
Y	N	N	2.3%
Y	N	Y	0.4%
Y	Y	N	2.7%
N	Y	Y	10.6%
Y	Y	Y	70.3%

[1] "N" is not taking multivitamins and "Y" is taking multivitamins.

Supplementary Table S2. Participant characteristics in the full sample [1], the complete case sample [2] for the outcomes of child neurodevelopment at 2 years of age in the 3D Cohort Study.

Characteristics	Complete case sample [1], n (%)	Full sample [2], n (%)	P
**Total**	1083	1534	
**Mother's age (years)**			0.71
<25	54 (5)	85 (5.6)	
25–<35	812 (75)	1128 (73.7)	
≥35	217 (20)	318 (20.8)	
**Maternal education**			0.41
Secondary school or less	54 (5)	94 (6.2)	
College	260 (24)	390 (25.6)	
Undergraduate university degree	467 (43.1)	628 (41.2)	
Graduate university studies	302 (27.9)	412 (27)	
**Household income (CAD)**			0.42
<30,000	67 (6.2)	116 (7.8)	
30,000–59,999	174 (16.1)	254 (17.2)	
60,000–79,999	189 (17.5)	262 (17.7)	
80,000–99,999	254 (23.5)	332 (22.4)	
≥100,000	399 (36.8)	515 (34.8)	
**Marital status**			0.51
Married	418 (38.6)	614 (40.1)	
Common law/partner	626 (57.8)	855 (55.8)	
Others	39 (3.6)	64 (4.2)	
**Prepregnancy BMI (kg/m^2)**			0.97
Underweight (<18.5)	64 (5.9)	90 (6.2)	
nomral weight (18.5–24.9)	707 (65.3)	953 (65.4)	
overweight (25–29.9)	189 (17.5)	245 (16.8)	
obese (≥30)	123 (11.4)	169 (11.6)	
**Parity**			0.80
0	634 (58.5)	890 (58)	
≥1	449 (41.5)	644 (42)	
**Mother born in Canada**			**0.01**
No	260 (24)	434 (28.3)	
Yes	823 (76)	1098 (71.7)	
**Caucasian**			**0.02**
No	177 (16.3)	305 (19.9)	
Yes	906 (83.7)	1226 (80.1)	
**Smoking during pregnancy**			0.57
No	947 (87.4)	1327 (86.7)	
Yes	136 (12.6)	204 (13.3)	

**Sex of the child**			0.84
Female	543 (50.1)	759 (49.7)	
Male	540 (49.9)	767 (50.3)	

BMI: Body Mass Index

[1] Complete case sample refers to the sample with complete data on diet quality, supplement intake, child neurodevelopmental outcomes (cognitive/motor/language), and all covariates.

[2] Full sample refers the sample with information on diet and supplement.

Supplementary Table S3-1. Regression coefficients of the adjusted models for child cognitive and language development at 2 years of age.

Parameters	Cognitive			Language		
	Regression coefficients	95% CI	P	Regression coefficients	95% CI	P
**Diet quality**						
High (HEI-C score ≥62.7)	4.2	(0.1, 8.2)	0.04	11.3	(3.1, 19.5)	0.01
Low (HEI-C score <62.7)	Ref.			Ref.		
**Multivitamins intake**						
Yes	3.0	(0.3, 5.8)	0.03	4.9	(-0.7, 10.4)	0.09
No	Ref.			Ref.		
**Diet quality*Multivitamins intake**	-5.2	(-9.4, -0.9)	0.02	-10.1	(-18.7, -1.4)	0.02
**Mother's age**						
<25	-0.2	(-3.3, 2.9)	0.89	-2.0	(-8.5, 4.6)	0.56
25–<35	Ref.			Ref.		
≥35	-1.6	(-3.2, 0.1)	0.06	-3.8	(-7.2, -0.4)	0.03
**Maternal education**						
Secondary school or less	Ref.			Ref.		
College	3.3	(0.1, 6.5)	0.05	3.6	(-3.3, 10.5)	0.30
Undergraduate degree	6.4	(3.2, 9.6)	<0.005	11.9	(5, 18.7)	<0.005
Graduate studies	7.0	(3.7, 10.4)	<.0001	11.1	(3.9, 18.2)	<0.005
**Household income (CAD)**						
<30,000	Ref.			Ref.		
30,000–59,999	3.4	(0.3, 6.6)	0.03	9.0	(2.3, 15.6)	0.01
60,000–79,999	5.2	(2, 8.5)	<0.005	9.8	(2.9, 16.7)	0.01
80,000–99,999	5.9	(2.6, 9.1)	<0.005	11.1	(4.3, 18)	<0.005
≥100,000	5.4	(2.2, 8.6)	<0.005	10.5	(3.8, 17.2)	<0.005
**Smoking during pregnancy**						
No	0.3	(-1.7, 2.3)	0.75	-2.5	(-6.7, 1.7)	0.24
Yes	Ref.			Ref.		
**Marital status**						
Married	Ref.			Ref.		
Common law/partner	2.2	(0.7, 3.7)	0.01	0.3	(-2.8, 3.4)	0.84
Others	2.8	(-1.1, 6.6)	0.16	7.0	(-1.1, 15)	0.09
**Prepregnancy BMI (kg/m^2)**						
Underweight (< 18.5)	0.7	(-2.1, 3.5)	0.63	-1.9	(-7.6, 3.7)	0.50

Normal weight (18.5–24.9)	Ref.			Ref.		
Overweight (25–29.9)	-0.9	(-2.6, 0.9)	0.34	-3.4	(-7, 0.2)	0.06
Obese (≥30)	-0.1	(-2.3, 2)	0.90	-1.1	(-5.5, 3.3)	0.62
**Parity**						
0	Ref.			Ref.		
≥1	-0.9	(-2.3, 0.5)	0.19	-2.2	(-5, 0.6)	0.13
**Mother born in Canada**						
No	-1.1	(-3.2, 1)	0.29	-6.3	(-10.5, -2.1)	<0.005
Yes	Ref.			Ref.		
**Caucasian**						
No	-2.1	(-4.4, 0.3)	0.09	-2.9	(-7.7, 2)	0.25
Yes	Ref.			Ref.		
**Sex of the child**						
Female	2.9	(1.6, 4.2)	<.0001	6.4	(3.8, 9.1)	<.0001
Male	Ref.			Ref.		
**Age at measurement (per year)**	-0.1	(-0.4, 0.2)	0.38	3.3	(2.7, 3.9)	<.0001
**Maternal calories intake (per 100 kcal)**	0.1	(0, 0.3)	0.06	0.03	(-0.3, 0.3)	0.84

HEI-C: Healthy Eating Index-Canada; CI: Confidence Interval; BMI: Body Mass Index

Supplementary Table S3-2. Regression coefficients of the adjusted models for fine and gross motor development.

Parameters	Fine motor			Gross motor		
	Regression coefficients	95% CI	P	Regression coefficients	95% CI	P
**Diet quality**						
High (HEI-C score ≥62.7)	0.5	(-0.5, 1.5)	0.31	0.7	(-0.2, 1.5)	0.13
Low (HEI-C score <62.7)	Ref.			Ref.		
**Multivitamins intake**						
Yes	0.2	(-0.5, 0.8)	0.62	-0.2	(-0.8, 0.4)	0.43
No	Ref.			Ref.		
**Diet quality*Multivitamins intake**	-0.7	(-1.8, 0.3)	0.17	-0.7	(-1.6, 0.3)	0.16
**Mother's age (years)**						
<25	0.02	(-0.7, 0.8)	0.95	-0.4	(-1.1, 0.2)	0.21
25–<35	Ref.			Ref.		
≥35	-0.2	(-0.6, 0.2)	0.40	-0.4	(-0.8, -0.1)	0.02
**Maternal education**						
Secondary school or less	Ref.			Ref.		
College	0.5	(-0.2, 1.3)	0.18	0.3	(-0.4, 1)	0.40
Undergraduate degree	1.1	(0.3, 1.9)	0.01	0.4	(-0.3, 1.1)	0.23
Graduate studies	1.1	(0.3, 1.9)	0.01	0.2	(-0.5, 1)	0.51
**Household income (CAD)**						
<30,000	Ref.			Ref.		
30,000–59,999	0.3	(-0.5, 1)	0.48	0.02	(-0.6, 0.7)	0.95
60,000–79,999	0.5	(-0.3, 1.3)	0.18	0.5	(-0.2, 1.2)	0.19
80,000–99,999	0.6	(-0.2, 1.3)	0.17	0.4	(-0.3, 1)	0.31
≥100,000	0.5	(-0.3, 1.3)	0.2	0.7	(0, 1.4)	0.04
**Smoking during pregnancy**						
No	0.1	(-0.4, 0.6)	0.73	-0.1	(-0.5, 0.4)	0.82
Yes	Ref.			Ref.		
**Marital status**						
Married	Ref.			Ref.		
Common law/partner	0.4	(0, 0.8)	0.03	0.4	(0.1, 0.7)	0.01
Others	0.1	(-0.9, 1)	0.87	1.1	(0.2, 1.9)	0.01
**Prepregnancy BMI (kg/m^2)**						
Underweight (< 18.5)	0.7	(0, 1.4)	0.04	-0.8	(-1.3, -0.2)	0.01
Normal weight (18.5–24.9)	Ref.			Ref.		
Overweight (25–29.9)	-0.2	(-0.6, 0.3)	0.48	-0.3	(-0.7, 0.04)	0.08
Obese (≥30)	-0.4	(-1, 0.1)	0.1	0.1	(-0.3, 0.6)	0.63

**Parity**						
0	Ref.			Ref.		
≥1	0.5	(0.1, 0.8)	0.01	0.5	(0.2, 0.8)	0.00
**Mother born in Canada**						
No	0.3	(-0.2, 0.8)	0.20	0.4	(0, 0.9)	0.07
Yes	Ref.			Ref.		
**Caucasian**						
No	-0.2	(-0.8, 0.4)	0.46	0.2	(-0.3, 0.7)	0.35
Yes	Ref.			Ref.		
**Sex of the child**						
Female	0.9	(0.5, 1.2)	<.0001	0.3	(0, 0.6)	0.04
Male	Ref.			Ref.		
**Age at measurement (per year)**	0.04	(0, 0.1)	0.38	0.03	(-0.03, 0.09)	0.32
**Maternal calories intake (per 100kcal)**	0.04	(-0.04, 0.10)	0.33	0.01	(-0.02, 0.04)	0.43

HEI-C: Healthy Eating Index-Canada; CI: Confidence Interval; BMI: Body Mass Index

Supplementary Table S4. Sensitivity analyses with imputation of missing values in covariates.

	Complete case sample			Imputed sample		
	**Regression coefficients**	**Standard error**	**P**	**Regression coefficients**	**Standard error**	**P**
**Cognitive, n**	1066			1182		
**Diet quality**						
High (HEI-C score ≥62.7)	4.16	2.06	0.044	4.94	2.00	0.013
Low (HEI-C score <62.7)	Ref.			Ref.		
**Multivitamins intake**						
Yes	3.02	1.40	0.031	3.08	1.36	0.023
No	Ref.			Ref.		
**Diet quality*Multivitamins intake**	-5.16	2.17	0.018	-5.83	2.10	0.005
**Language, n**	981			1108		
**Diet quality**				Ref.		
High (HEI-C score ≥62.7)	11.31	4.19	0.007	10.77	4.00	0.007
Low (HEI-C score <62.7)	Ref.			Ref.		
**Multivitamins intake**						
Yes	4.87	2.83	0.086	5.24	2.73	0.055
No	Ref.			Ref.		
**Diet quality*Multivitamins intake**	-10.07	4.42	0.023	-9.99	4.21	0.018
**Fine Motor, n**	1040			1134		
**Diet quality**				Ref.		
High (HEI-C score ≥62.7)	0.52	0.51	0.314	0.40	0.50	0.422
Low (HEI-C score <62.7)	Ref.			Ref.		
**Multivitamins intake**						
Yes	0.17	0.35	0.623	0.12	0.34	0.717
No	Ref.			Ref.		
**Diet quality*Multivitamins intake**	-0.74	0.54	0.170	-0.65	0.52	0.215
**Gross motor, n**	1040			1134		
**Diet quality**				Ref.		
High (HEI-C score ≥62.7)	0.68	0.44	0.128	0.63	0.44	0.147
Low (HEI-C score <62.7)	Ref.			Ref.		
**Multivitamins intake**						
Yes	-0.24	0.30	0.432	-0.34	0.30	0.246
No	Ref.			Ref.		
**Diet quality*Multivitamins intake**	-0.65	0.47	0.163	-0.60	0.46	0.193

HEI-C: Healthy Eating Index-Canada

Supplementary Table S5. Association between multivitamin use and omega-3 supplement use.

	Omega-3 use	No omega-3 use	P for chi-square
	n (%)	n (%)	
Multivitamin use	90 (6.6)	1279 (93.4)	0.06
No multivitamin use	5 (3.0)	162 (97.0)	

Supplementary Table S6. Association between taking omega-3 and neurodevelopment outcomes.

	Omega-3 use	No omega-3 use	P for t-test
	mean(SD)	mean(SD)	
Whole sample			
Cognitive	100.4 (11.4)	101.4 (10.9)	0.46
Language	54.8 (23.6)	58.3 (23.4)	0.23
Fine motor	11.6 (2.7)	11.7 (2.6)	0.78
Gross motor	9.0 (2.3)	8.7 (2.0)	0.28
Sample with low diet quality			
Cognitive	100.2 (11.4)	99.8 (9.4)	0.86
Language	53.2 (22.9)	58.0 (23.1)	0.26
Fine motor	11.6 (2.7)	11.6 (2.9)	0.94
Gross motor	8.9 (2.3)	8.8 (1.9)	0.86

Supplementary Table S7. Mean (SD) intake of selected nutrients in the study population.

	Mean (SD)			
Nutrients	Lower HEI-C, no multivitamins	Higher HEI-C, no multivitamins	Lower HEI-C, took multivitamins	Higher HEI-C, took multivitamins
N	98	69	669	698
Iron (mg)	14.82 (4.26)	15.29 (3.8)	45.77 (22.28)	48.46 (24.16)
Manganese (mg)	315.39 (88.47)	393.34 (97.96)	375.41 (89.58)	460.06 (104.66)
Potassium (mg)	2978.15 (748.65)	3627.01 (796.88)	3073.15 (747.82)	3680.93 (816.9)
Zinc (mg)	11.1 (3.31)	11.67 (2.65)	19.81 (5.01)	21.2 (5.23)
Choline (mg)	178.68 (54.99)	201.72 (63.55)	182.93 (62.29)	207.3 (61.68)
Folate (µg)	233.82 (73.28)	290.57 (84.06)	1783.52 (1343.87)	1900.39 (1397.05)
Vitamin A in RAE(µg)	783.38 (288.49)	1002.2 (914.97)	1756.51 (732.27)	1893.13 (574.66)
Vitamin B1 (mg)	1.9 (0.67)	1.86 (0.56)	3.76 (3.17)	3.78 (2.21)
Vitamin B2 (mg)	2.28 (0.67)	2.2 (0.52)	4.19 (3.16)	4.28 (2.28)
Vitamin B3 (mg)	21.01 (5.82)	22.22 (4.81)	38.59 (7.65)	40.67 (7.83)
Vitamin B6 (mg)	1.62 (0.44)	2 (0.46)	5.69 (6.11)	6.17 (5.13)
Vitamin B12 (µg)	4.53 (1.85)	5.79 (3.98)	12.64 (49.86)	11.48 (19.58)
Vitamin C (mg)	135.34 (71.33)	202.36 (86.93)	236.13 (117.12)	279.5 (94.85)
Vitamin D (µg)	5.09 (2.62)	5.8 (3.2)	13.94 (4.99)	15.34 (9.1)

SD: standard deviation; HEI-C: Healthy Eating Index-Canada; RAE: Retinol Activity Equivalent
